# A153 IMPLEMENTATION OF THE CHAMPLAIN REGION GASTROINTESTINAL ENDOSCOPY CENTRAL INTAKE (GICI) PROGRAM

**DOI:** 10.1093/jcag/gwad061.153

**Published:** 2024-02-14

**Authors:** A Monod, M Jarrar, S Coates, H Burns, R Prihar, C Dubé

**Affiliations:** Ottawa University, Ottawa, ON, Canada; Queensway Carleton Hospital, Ottawa, ON, Canada; Queensway Carleton Hospital, Ottawa, ON, Canada; Queensway Carleton Hospital, Ottawa, ON, Canada; Queensway Carleton Hospital, Ottawa, ON, Canada; Ottawa University, Ottawa, ON, Canada

## Abstract

**Background:**

The Champlain region of Ontario is implementing a regional central intake triage program for screening colonoscopy services (FIT+, screening and surveillance indications). The aim of this program is to improve the delivery of regional endoscopy services, so that access to resource-intensive endoscopy procedures can be equitable, timely and evidence-based. We characterized the first phase of implementation of the Champlain Regional GI Central Intake (GICI).

**Aims:**

The primary objective of this study is to identify program strengths and opportunities for quality improvement.

**Methods:**

Prospective data was collected from August 1, 2022 to August 31, 2023. Descriptive statistics were analyzed. Discrete data were presented in terms of counts, percentages and means where appropriate.

**Results:**

1942 CIP referrals were received during the period of study. 1478(76.1%) referrals were submitted by fax, and 571(29.4%) were for positive FITs. 703(36.2%) referrals were declined, with 374(19.3%) not meeting Cancer Care Ontario (CCO) referral indications, 84(4.3%) being duplicates and 56(2.9%) being symptomatic. Prior to a policy change in March 2023, 23(1.2%) referrals were declined due to incomplete referral forms. 581(29.9%) referrals indicated a preferred physician or hospital site. On average, triage decisions were made within 11.4 hours of receipt of referrals for positive FIT, and 6.8 days for other indications. The average time from FIT positivity to referral receipt at CIP was 20.0 days. In the first quarter of 2023, 75th percentile wait times for colonoscopy were 35 days for FIT positivity, 150 days for symptoms, and 346 days for other screening indications.

**Conclusions:**

GICI and the use of CCO evidence-based guidance reduces the need for colonoscopy in nearly 20% of referrals. It also decreases physician administrative burdens by eliminating duplicate referrals. Referrals should not be declined due to incomplete forms. Measures to improve timely access to colonoscopy for symptomatic patients should be explored. Most referred patients do not have a preferred physician or hospital site. Further primary care support and engagement is needed to encourage the use of the GICI as well as immediate referral for FIT+ colonoscopy.

**Funding Agencies:**

None

